# Transplantation of dental pulp stem cells improves long-term diabetic polyneuropathy together with improvement of nerve morphometrical evaluation

**DOI:** 10.1186/s13287-017-0729-5

**Published:** 2017-12-13

**Authors:** Maiko Omi, Masaki Hata, Nobuhisa Nakamura, Megumi Miyabe, Shogo Ozawa, Hitoshi Nukada, Masami Tsukamoto, Kazunori Sango, Tatsuhito Himeno, Hideki Kamiya, Jiro Nakamura, Jun Takebe, Tatsuaki Matsubara, Keiko Naruse

**Affiliations:** 1 Department of Removable Prosthodontics, School of Dentistry, Aichi Gakuin University, 2-11 Suemori-dori, Chikusa-ku, Nagoya, Aichi 464-8651 Japan; 2 Department of Internal Medicine, School of Dentistry, Aichi Gakuin University, 2-11 Suemori-dori, Chikusa-ku, Nagoya, Aichi 464-8651 Japan; 3 Department of Medicine, University of Otago Medical School, PO Box 913, Great King Street, Dunedin, New Zealand; 4 Laboratory of Peripheral Nerve Pathophysiology, Department of Sensory and Motor Systems, Tokyo Metropolitan Institute of Medical Science, 2-1-6 Kamikitazawa, Setagaya-ku, Tokyo, 156-8506 Japan; 5 Division of Diabetes, Department of Internal Medicine, Aichi Medical University School of Medicine, Nagakute, Aichi Japan

**Keywords:** Dental pulp stem cells, Diabetic polyneuropathy, Cell transplantation, Nerve repair

## Abstract

**Background:**

Although previous reports have revealed the therapeutic potential of stem cell transplantation in diabetic polyneuropathy, the effects of cell transplantation on long-term diabetic polyneuropathy have not been investigated. In this study, we investigated whether the transplantation of dental pulp stem cells (DPSCs) ameliorated long-term diabetic polyneuropathy in streptozotocin (STZ)-induced diabetic rats.

**Methods:**

Forty-eight weeks after STZ injection, we transplanted DPSCs into the unilateral hindlimb skeletal muscles. Four weeks after DPSC transplantation (i.e., 52 weeks after STZ injection) the effects of DPSC transplantation on diabetic polyneuropathy were assessed.

**Results:**

STZ-induced diabetic rats showed significant reductions in the sciatic motor/sensory nerve conduction velocity, increases in the current perception threshold, and decreases in capillary density in skeletal muscles and intra-epidermal nerve fiber density compared with normal rats, all of which were ameliorated by DPSC transplantation. Furthermore, sural nerve morphometrical analysis revealed that the transplantation of DPSCs significantly increased the myelin thickness and area. DPSC-conditioned media promoted the neurite outgrowth of dorsal root ganglion neurons and increased the viability and myelin-related protein expression of Schwann cells.

**Conclusions:**

These results indicated that the transplantation of DPSCs contributed to the neurophysiological and neuropathological recovery from a long duration of diabetic polyneuropathy.

**Electronic supplementary material:**

The online version of this article (doi:10.1186/s13287-017-0729-5) contains supplementary material, which is available to authorized users.

## Background

Diabetic polyneuropathy is characterized by high morbidity and various symptoms. The major pathophysiological alterations of diabetic polyneuropathy are the degeneration of the nerves and abnormal microvasculature in the nerves [1–3]. The impairment of small nerve fibers causes sensational disorder, whereas impairment of large fibers leads to a reduction in nerve conduction and loss of tactile perception [4]. Currently, drugs for painful diabetic polyneuropathy provide modest amelioration from the symptoms, but there are few drugs that have been convincingly shown to slow the progression of diabetic polyneuropathy. The final goal of treatment for diabetic polyneuropathy is to improve nerve function and pathological abnormalities with management of the neuropathic pain and reduced risk of foot ulceration and amputation [5–7]. Therefore, more effective treatments for diabetic polyneuropathy are needed.

We and others have previously demonstrated that the transplantation of cultured endothelial progenitor cells (EPCs) and mesenchymal stem cells (MSCs), including dental pulp stem cells (DPSCs), improved the reduction of nerve conduction velocity and nerve blood flow, and intra-epidermal nerve fiber density (IENFD) [8–12]. We also demonstrated that the transplantation of bone marrow-derived mononuclear cells (BM-MNCs) alleviated hyperalgesia in the early stage of diabetes in rats [13]. However, most previous studies were of relatively short duration (the duration of diabetes was 2–16 weeks). The morphometric analysis of myelinated fibers in sural nerves did not show significant differences in most components except for axonal circularity in rats with a 12-week duration of streptozotocin (STZ)-induced diabetes [9, 14]. These results suggest that we need to evaluate the effects of cell transplantation in diabetic animals with long-term diabetes.

In the present study, we examined diabetic rats for 48 weeks after STZ injection to demonstrate whether or not the transplantation of DPSCs ameliorates diabetic polyneuropathy with morphological improvement of the peripheral nerves. Here, we found, for the first time, that the intramuscular transplantation of DPSCs ameliorated long-term diabetic polyneuropathy, accompanied by neurophysiological and morphological recovery of the peripheral nerves.

## Methods

### Animals

Male Sprague-Dawley (SD) rats at 6 weeks of age were obtained from Chubu Kagakushizai (Nagoya, Japan). For the induction of diabetes, STZ (Sigma Chemical Co., St. Louis, MO, USA) (60 mg/kg body weight) was intraperitoneally injected into rats. Blood glucose levels greater than 14 mmol/l were identified as diabetes at 1 week after STZ injection and were used in the experiments. Small doses of insulin pellet (LinShin Canada, Inc. Toronto, Canada) were administered subcutaneously once a month from 8 weeks after STZ injection to avoid excess hyperglycemia (Fig. 2a). Age-matched male SD rats were used as control. Rats were housed in a room maintained under controlled temperature (24 ± 1.0 °C) on a 12-h light/dark cycle and were given standard laboratory rat chow with water ad libitum. All experimental protocols were approved by the Institutional Animal Care and Use Committees of Aichi Gakuin University (AGUD 059) and were conducted in accordance with the United States Public Health Service’s Policy on Humane Care and Use of Laboratory Animals. All efforts were made to minimize animal suffering.

### Isolation and culture of DPSCs

Dental pulp tissue was excised from the incisor teeth of 6-week-old male SD rats, and DPSCs were isolated and cultured in an alpha modification of the Eagle’s medium (α-MEM) (GIBCO Lab Inc., Grand Island, NY) with 20% fetal bovine serum (FBS; GIBCO) as previously reported [12]. DPSCs from passage 3 or 4 were used for all experiments.

### Characterization of DPSCs

Cells were stained with the R-PE-conjugated antibodies against rat CD29 (Becton Dickinson, Franklin Lakes, NJ, USA), CD90, CD45 (Becton Dickinson), and CD34 (Santa Cruz Biotechnology, Santa Cruz, CA, USA), and the FITC-conjugated antibody against rat CD49d to characterize the DPSCs by flow cytometry (Miltenyi Biotec, Bergisch Gladbach, Germany). Isotype-identical antibodies served as the controls. Data were analyzed with MACSquant software (Miltenyi Biotec). The multi-differentiability of DPSCs was assessed by their differentiation into osteoblasts, chondrocytes, and adipocytes according to the manufacturer’s instructions (R&D Systems, Minneapolis, MN, USA).

### Transplantation of DPSCs

Forty-eight weeks after the STZ induction of diabetes, the rats were anesthetized with pentobarbital (50 mg/kg, intraperitoneally) and underwent transplantation of DPSCs into the hind limb skeletal muscles. The DPSCs (1 × 10^6^ cells) were suspended in 1.0 ml saline and injected into 10 points in the unilateral gastrocnemius, soleus, and biceps femoris muscles of both the normal and the diabetic rats using a 26-gauge needle. Saline was injected into the opposite side of hind limb skeletal muscles as the control. The parameters discussed below were measured 4 weeks after transplantation.

### Sciatic nerve conduction velocities

Rats were anesthetized by isoflurane inhalation, and the near nerve temperature was maintained at 37 °C by a warming pad using a multipurpose thermometer (Bioresearch Co., Nagoya, Japan). The motor nerve conduction velocity (MNCV) and sensory nerve conduction velocity (SNCV) in the sciatic nerve were measured using a Neuropak MEB-9400 (Nihon-Koden, Osaka, Japan).

### Sciatic nerve blood flow

Rats were deeply anesthetized by isoflurane inhalation and sciatic nerve blood flow (SNBF) was measured using a Laser Doppler Blood Flow Meter (FLO-N1; Omega Wave Inc., Tokyo, Japan) as previously described [12]. During the procedure, the rats were laid out on a heated pad and the near nerve temperature was maintained at 37 °C using a thermometer (Bioresearch Co.).

### Current perception threshold using a Neurometer

The current perception threshold (CPT) of the sensory nerve fibers was measured in diabetic and normal rats using a CPT/LAB neurometer (Neurotron, Denver, CO, USA). Each rat was kept in an awake state in a Ballman cage (Natsume, Tokyo, Japan). The plantar surfaces of the rats were stimulated by 5, 250, and 2000 Hz sine-wave pulses. The intensity of each stimulation was gradually increased automatically. The minimum intensity when startled was defined as the current perception threshold of each rat.

### Capillary density in the hind limb skeletal muscles

Rats were killed with an overdose of pentobarbital (150 mg/kg), and the hind limb skeletal muscles were fixed in a 4% paraformaldehyde solution. The fixed materials were embedded in paraffin and cut into 5-μm sections. The sections were incubated overnight at 4 °C with the anti-von Willebrand factor polyclonal antibody (DAKO Japan, Tokyo, Japan) and subsequently stained using the Simplestain rat system (Nichirei, Tokyo, Japan). The capillary endothelial cells were counted under light microscopy (Leica Microsystems, Wetzlar, Germany).

### Laser Doppler perfusion image of the hind limb blood flow

Rats were anesthetized and placed on a heating pad to keep a constant rectal temperature of 37 °C. Hind limb blood flow was visualized using a laser Doppler perfusion image (LDPI) analyzer (Moor Instruments, Devon, UK). Low to no flow was displayed as dark blue, whereas high flow was displayed as red.

### Intra-epidermal nerve fiber density

After the fixation of the footpads, tissues were immersed in an OCT compound (Sakura Finetechnical, Tokyo, Japan) containing liquid nitrogen and isopentane. Three longitudinal 25-μm thick footpad sections from each rat were cut on a cryostat (Leica CM 1510 S). The sections were incubated overnight at 4 °C with the primary antibody (anti-PGP9.5 antibody; Millipore, Tokyo, Japan). Alexa Fluor 594-coupled goat anti-mouse IgG antibody (Invitrogen, Carlsbad, CA, USA) was applied as the second antibody. IENFD was assessed as previously reported [13]. Nerve fibers were counted blindly by three independent investigators under an FV10i confocal system (Olympus, Tokyo, Japan) and the average values were used.

### mRNA expression of the hind limb skeletal muscles

Total RNA was extracted from the frozen samples of hind limb skeletal muscles and purified using an RNeasy kit (QIAGEN, Valencia, CA, USA) according to the manufacturer’s instructions. cDNA was synthesized using ReverTra Ace (Toyobo, Osaka, Japan). Primers and probes for *fibroblast growth factor 2* (*FGF2*; also known as *bFGF*), *nerve growth factor* (*NGF*), *neurotrophin 3* (*NT-3*) and *β*_*2*_ *microglobulin* (Applied Biosystems, Foster City, CA, USA) were purchased. Real-time quantitative polymerase chain reaction (PCR) was performed using the ABI Prism 7000 (Applied Biosystems) and calculated by the ΔΔC^t^ method.

### Morphometrical analysis of the sural nerves

The sural nerves were fixed in 2% glutaraldehyde followed by osmium tetroxide and were embedded in Epon. Semi-thin sections (0.5-μm thick sections) were stained with toluidine blue and examined by light microscopy (Leica microsystems). The total complement of sural nerve myelinated fibers was assessed using the analysis software ImageJ (Research Services Branch of the National Institutes of Mental Health, Bethesda, MD, USA). The investigator was blinded to the group identity throughout the morphometric process.

### Preparation of DPSC-conditioned media (DPSC-CM)

When DPSCs reached 70% confluence in 10-cm dishes, they were maintained in Dulbecco’s modified Eagle’s medium (DMEM) containing penicillin and streptomycin in a 5% CO_2_ humidified atmosphere at 37 °C. After 24 h, the culture media were collected, concentrated 10 times using 10-kDa centrifugal filters (Amicom Ultra-15, Nihon Millipore, Tokyo, Japan), and frozen at −20 °C until use.

### Primary culture of dorsal root ganglion (DRG) neurons and evaluation of neurite outgrowth

DRG neuron cultures were prepared from 8-week-old male SD rats as previously described [15]. DRG neurons were cultured for 24 h in serum-free medium (DMEM/F12 supplemented with B27 (Invitrogen)) for use in neurite outgrowth with DPSC-CM. DRG neurons were immunostained with rabbit polyclonal anti-neurofilament heavy-chain antibody (Millipore). Alexa Fluor 488-coupled goat anti-rabbit IgG antibody (Invitrogen) was applied as the second antibody. Coverslips were counterstained with 4’ ,6-diamidino-2-phenylindole (DAPI; Millipore). Neurite outgrowth was observed in 40–50 neurons per cover slip, and the total length and joint number of neurites was calculated by a computed image analysis system (Angiogenesis Image Analyzer Ver. 2, KURABO Industries, Osaka, Japan).

### Cell viability assay in immortalized adult Fischer rat Schwann cells, IFRS1

IFRS1 cells, immortalized adult rodent Schwann cells, were seeded on plastic dishes and were maintained in DMEM containing 5% FBS, 20 ng/ml recombinant human heregulin-β (Upstate, Lake Placid, NY, USA), and 5 μM forskolin (Sigma) [16, 17]. IFRS1 cells were seeded in 96-well plates (5 × 10^3^ cells/well), and were maintained in DMEM containing 1% FBS media. After 12 h, the cells were incubated with DPSC-CM, and the cell viability in 1-, 3-, and 5-day culture was assessed by the CCK-8 assay (Dojin Laboratories, Kumamoto, Japan). The absorbance at 450 nm of each well was read on a spectrophotometer. Three independent experiments were performed in quadruplicate.

### Analysis of myelin-related protein formation in IFRS1 cells

IFRS1 cells were cultured with DPSC-CM in DMEM containing 1% FBS supplemented with 50 μg/ml ascorbic acid for 5 days. Western blot analyses were performed with the rabbit anti-myelin protein zero (MP0) polyclonal antibody (Abcam) and rabbit anti-β-actin monoclonal antibody (Cell Signaling Technologies, Beverly, MA, USA).

For immunostaining, 4% PHA-fixed cells were incubated with a 1:400 dilution of the MP0 substrate antibody. Alexa Fluor 568-coupled donkey anti-rabbit IgG antibody (1:200; Invitrogen) was applied at room temperature for 1 h. Conventional microscopy images were taken using the FV10i confocal system (Olympus).

### Statistical analysis

All group values are expressed as the mean ± standard error of the mean (SEM). Statistical analyses were made by Student’s *t* test for comparisons between two groups and one-way analysis of variance (ANOVA) followed by the Bonferroni correction for multiple comparisons.

## Results

### Characterization in normal and diabetic rats

The diabetic rats kept the high glucose during the experimental periods (Fig. 2b). The diabetic rats at 52-weeks after the STZ injection exhibited reduced body weight and significantly increased plasma glucose levels compared with normal rats (normal rats: body weight 589.1 ± 14.1 g; blood glucose 5.3 ± 0.8 mmol/l (*n* = 10); diabetic rats: body weight 435.4 ± 44.7 g; blood glucose 18.2 ± 0.7 mmol/l (*n* = 7)).

### Identification of DPSCs

**Fig. 1:**
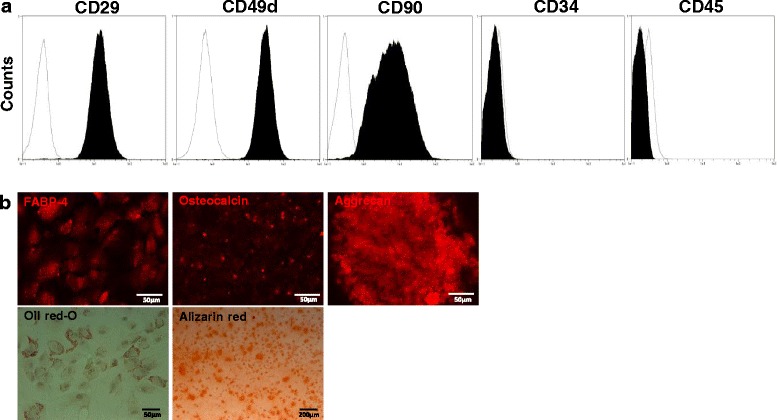
Characterization and differentiation of DPSCs. **a** DPSCs derived from 6-week-old Sprague-Dawley rats were positive for mesenchymal stem cell markers (CD29, CD49d, CD90) and negative for hematopoietic markers (CD34 and CD45) (*black area*). Isotype-identical antibodies served as the controls (*white area*). **b** DPSCs could differentiate into adipogenic, osteogenic, and chondrogenic lineages in vitro. For discrimination, Oil Red O and fatty acid-binding protein-4 were used for adipocytes, Alizarin Red and osteocalcin for osteoblasts, and aggrecan for chondrocytes

DPSCs showed high expression of CD29, CD90, and CD49d, which are common stem cell markers in MSCs. DPSCs lacked the expression of CD34 and CD45 (Fig. 1a). DPSCs differentiated into adipocytes, osteoblasts, and chondrocytes by each induction. The cells induced into chondrocytes showed stainability in Aggrecan. Adipogenesis was assessed by staining with Oil Red O and fatty acid-binding protein-4. DPSCs were also differentiated into osteoblasts stained with osteocalcin, and showed marked mineralization by Alizarin Red S staining (Fig. 1b).

### DPSC transplantation ameliorates MNCV, SNCV, and SNBF in diabetic rat*s*

**Fig. 2:**
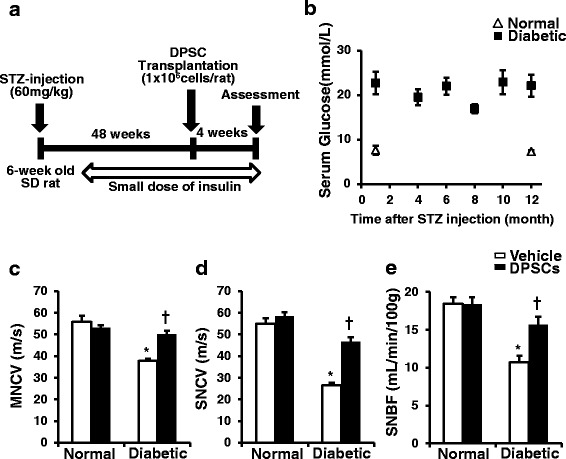
Experimental animal protocol and clinical and neurophysiological measurements. **a** Dental pulp stem cells (*DPSCs*) were transplanted into unilateral hind limb skeletal muscles 48 weeks after streptozotocin (*STZ*) injection, and neurophysiological assessments were performed 4 weeks after DPSC transplantation. Small doses of insulin pellet were administered subcutaneously once a month from 8 weeks after STZ injection to avoid excess hyperglycemia. **b** Blood glucose concentrations during the experiment. Blood glucose levels were determined at the indicated time points. **c** Sciatic motor nerve conduction velocity (*MNCV*). MNCV was measured between the ankle and sciatic notch. **d** Sciatic sensory nerve conduction velocity (*SNCV*). SNCV was measured between the ankle and knee; *n* = 5. **e** Sciatic nerve blood flow (*SNBF*). SNBF was measured using a Laser Doppler Blood Flow Meter. The results are means ± SEM. *P < 0.01, vs. vehicle-injected side of normal rats; ^†^ *P* < 0.01, vs. vehicle-injected side of diabetic rats. *SD* Sprague-Dawley

On the vehicle-injected side of the diabetic rats, MNCV and SNCV were significantly reduced compared with the vehicle-injected side of the normal rats, which were ameliorated by DPSC transplantation (Fig. 2c and d) (*P* < 0.01 for both). SNBF was also significantly decreased on the vehicle-injected side of the diabetic rats compared with the vehicle-injected side of the normal rats (Fig. 2e) (*P* < 0.01). In the diabetic rats, DPSC transplantation increased SNBF compared with the vehicle-injected side (*P* < 0.01). Transplantation of DPSCs in the normal rats showed no significant changes in NCV or SNBF.

### Reduced sensory perception in the diabetic rats is ameliorated by DPSC transplantation

**Fig. 3:**
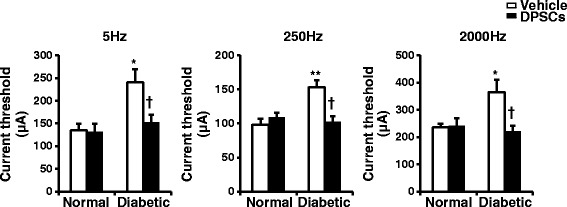
The functional integrity of the sensory nerve fibers was assessed by the perception threshold (CPT). The CPT was quantified at each frequency by stimulating Aβ fibers at 2000 Hz, Aδ fibers at 250 Hz, and C fibers at 5 Hz using the Neurometer; *n* = 5. The results are means ± SEM. *P < 0.05, **P < 0.01, vs. vehicle-injected side of normal rats; ^†^ *P* < 0.05, vs. vehicle-injected side of diabetic rats. *DPSCs* dental pulp stem cells

The CPT was quantified at each frequency by stimulating Aβ fibers at 2000 Hz, Aδ fibers at 250 Hz, and C fibers at 5 Hz, with an output intensity range of 0.01 to 9.99 mA. After 52 weeks of diabetes, CPTs at Aβ, Aδ, and C fibers were significantly increased compared with those in age-matched normal rats (Fig. 3). On the DPSC-transplanted side of the diabetic rats, those deteriorations in sensation were significantly improved compared with those on the vehicle-injected side. These results indicated that DPSC transplantation ameliorated hypoalgesia in the diabetic rats. On the other hand, the transplantation of DPSCs into the normal rats did not induce significant changes in the CPTs.

### DPSC transplantation increases the mRNA expression of NGF in the skeletal muscles of diabetic rats

**Fig. 4:**
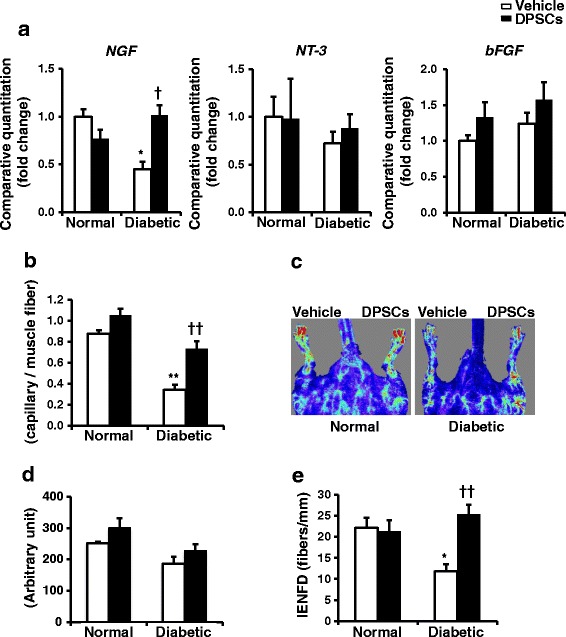
mRNA expressions, capillary density/blood flow in the hind limb and intra-epidermal nerve fiber density. **a** mRNA expression in the hind limb skeletal muscles. Four weeks after the transplantation of dental pulp stem cells (*DPSCs*) (the duration of diabetes was 52 weeks), the mRNA expression levels of nerve growth factor (*NGF*), neurotrophin 3 (*NT-3*), and basic fibroblast growth factor (*bFGF*) in the hind limb skeletal muscles were evaluated by real-time quantitative PCR. The results are means ± SEM; *n* = 7. *P < 0.05, vs. vehicle-injected side of normal rats; ^†^ *P* < 0.05, vs. vehicle-injected side of diabetic rats. **b** The capillary endothelial cells were stained with the anti-von Willebrand factor (vWF) polyclonal antibody. Quantitative analyses for the capillary/muscle fiber ratio of the vehicle-injected and DPSC-transplanted side of skeletal muscles in normal and diabetic rats. The results are means ± SEM. **P < 0.01, vs. vehicle-injected side of normal rats; ^††^ *P* < 0.01, vs. vehicle-injected side of diabetic rats. **c** Representative LDPI of the hind limb blood flow of rats. DPSC transplantation increased blood flow (*yellow* to *red* color) in the DPSC-injected side of the hind limb of diabetic rats. **d** Computer-assisted quantitative analyses of hind limb blood flow in normal and diabetic rats. **e** Intra-epidermal nerve fiber density (*IENFD*) was evident in both the epidermis and dermis of foot skin by fluorescent imaging for PGP9.5. Intra-epidermal nerve fiber profiles were counted blindly by three independent investigators and the average values were used. The results are means ± SEM. *P < 0.05, vs. vehicle-injected side of normal rats; ^††^ *P* < 0.01, vs. vehicle-injected side of diabetic rats; *n* = 7

The mRNA expression of NGF was significantly decreased in the skeletal muscles of diabetic rats compared with those of normal rats (Fig. 4a) (*P* < 0.05). Transplantation of DPSCs significantly increased NGF mRNA expression in diabetic rats (*P* < 0.05). The NT-3 and bFGF mRNA expression levels tended to increase on the DPSC-transplanted side of the diabetic rats, although those increases were not significant (Fig. 4a).

### DPSC transplantation increases the capillary density of skeletal muscles and hind limb blood flow in diabetic rats

Capillaries in skeletal muscles were visualized using immunostaining against von Willebrand factor, a specific marker for endothelial cells. Quantitative analyses revealed that the capillary/muscle fiber ratio in the diabetic rats was significantly reduced compared with that in the normal rats (*P* < 0.01) (Fig. 4b). In the diabetic rats, transplantation of DPSCs significantly increased the number of capillaries in skeletal muscles compared with that on the vehicle-injected side (vehicle-injected side: 0.34 ± 0.05; DPSC-injected side: 0.73 ± 0.07; *P* < 0.01). In the normal rats, the capillary/muscle fiber ratio was not significantly different between the vehicle-injected and DPSC-injected side. LPDI revealed that the transplantation of DPSCs tended to augment blood flow in the DPSC-injected side of the hind limb compared with that in the vehicle-injected side, although these were not significant (Fig. 4c and d).

### DPSC transplantation increased IENFD in the diabetic rats

Quantitative analyses revealed that IENFD was significantly reduced in the diabetic rats compared the normal rats (normal vehicle-injected side: 22.14 ± 2.38/mm; diabetic vehicle-injected side: 11.81 ± 1.64/mm; *P* < 0.05), which was significantly ameliorated by the DPSC transplantation (diabetic DPSC-injected side: 25.34 ± 2.25/mm; *P* < 0.01) (Fig. 4e). No significant difference was detected in the normal rats with transplanted DPSCs.

### DPSC-CM promotes neurite outgrowth of DRG neurons

**Fig. 5:**
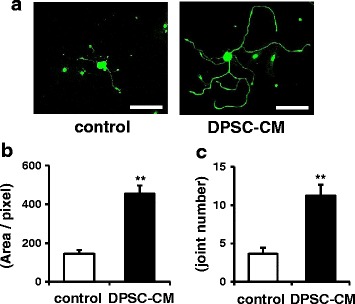
**a** Dental pulp stem cell-conditioned media (*DPSC-CM*) promoted neurite outgrowth of DRG neurons. DRG neurons (stained with neurofilament heavy-chain immunofluorescence) extended neurites, and the extensions were significantly promoted in the presence of DPSC-CM compared with that in the absence of DPSC-CM. *Scale bars* = 100 μm. The total length (**b**) and joint number (**c**) of neurite outgrowths in DRG neurons were calculated using a computed image analysis system (Angiogenesis Image Analyzer Ver. 2, KURABO Industries, Osaka, Japan). The results are means ± SEM. **P < 0.01, vs. control group

DRG neurons (stained with neurofilament heavy-chain immunofluorescence) showed extended neurites, and the extensions were significantly promoted in the presence of DPSC-CM compared with that in the absence of DPSC-CM (Fig. 5a). The total length and joint number of neurites in the presence of DPSC-CM were significantly increased by 3.2-fold and 3.1-fold, respectively, compared with those in the control (*P* < 0.01) (Fig. 5b and c).

### DPSC transplantation ameliorates the morphological defects of the sural nerves in diabetic rats

**Fig. 6:**
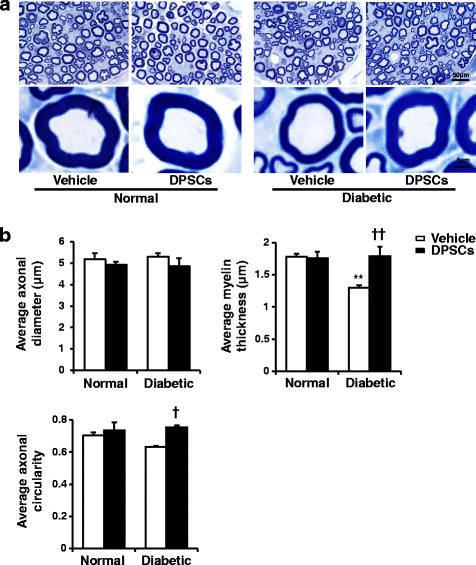
**a** Representative semi-thin cross-sections of the sural nerves of rats. Toluidine blue stained. **b** The morphometry of the myelinated nerve fibers. There were no significant differences in the diameter and circularity between the normal and diabetic rats. Myelin thickness was less in the diabetic rats compared with the normal rats. The transplantation of dental pulp stem cells (DPSCs) significantly increased the myelin thickness and circularity. The results are means ± SEM. **P < 0.01, vs. vehicle-injected side of normal rats; ^†^ *P* < 0.05, ^††^ *P* < 0.01, vs. vehicle-injected side of diabetic rats; *n* = 5

Myelinated fiber morphologic analyses of sural nerves are shown in Additional file 1 (Table S1) and Fig. 6a and b. The diabetic rats at 52-weeks after the STZ injection showed decreases in the fiber area, occupancy rate, myelin area, and myelin thickness, and an increase in the axonal-to-myelin area ratio, compared with the normal rats. There were no significant differences in the axonal area, diameter, and circularity between the normal and diabetic rats. Myelin area and myelin thickness were less by 52.5% and 27.1%, respectively, in the diabetic rats compared with the normal rats. The transplantation of DPSCs significantly increased the myelin thickness and area. According to this, other morphological defects of myelinated sural nerve fibers in diabetic rats were ameliorated by DPSC transplantation. These results indicated that the transplantation of DPSCs predominantly affected myelin formation in the sural nerve morphology.

### DPSC-conditioned media promotes Schwann cell viability and myelin formation

**Fig. 7:**
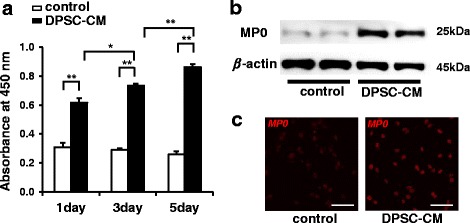
Dental pulp stem cell-conditioned media (*DPSC-CM*) promoted Schwann cell viability and myelin formation. **a** The effects of DPSC-CM on Schwann cell viability were measured by CCK-8 analysis. The results are means ± SEM. *P < 0.05, **P < 0.01; *n* = 12. **b** The protein expression of myelin protein zero (*MP0*), a related protein for the myelination of Schwann cells, was assessed after a 5-day incubation with DPSC-CM by Western blotting. **c** Microscopic analysis of MP0-positive myelin segments in Schwann cells treated with DPSC-CM further revealed the expression of MP0 compared with that in the control

The effects of DPSC-CM on Schwann cell viability were measured by CCK-8 analysis. When immortalized rodent Schwann cells (IFRS1) were cultured in the absence of DPSC-CM for 1, 3, and 5 days, IFRS1 cells failed to grow. By contrast, when cells were cultured in the presence of DPSC-CM for 1, 3, and 5 days, the CCK-8 values of IFRS1 cells were significantly increased in a time-dependent manner (Fig. 7a).

We examined the effect of DPSC-CM on the protein expression of MP0, which is critical for the myelination of nerve fibers, in IFRS1 cells after 5 days of incubation with DPSC-CM by Western blot and immunofluorescence staining. DPSC-CM significantly increased the expression of MP0 in IFRS1 cells (Fig. 7b). A microscopic analysis of MP0-positive myelin segments in IFRS1 cells treated with DPSC-CM further revealed the increased expression of MP0 (Fig. 7c).

## Discussion

The present study, for the first time, demonstrated that the transplantation of DPSCs improved diabetic polyneuropathy in long-term diabetic rats. DPSCs were transplanted into rats with 48-week diabetic duration and showed greater improvements in the sensory disorder, reduced MNCV/SNCV, SNBF, capillary density in skeletal muscles, and IENFD in diabetic rats. Morphometric analyses suggested that the effects of DPSC transplantation occurred mainly through the increased myelin thickness and area in the sural nerves.

Most of the previous studies on cell therapy for diabetic polyneuropathy used young animals with relatively short diabetic duration (2–20 weeks). Although we and others have demonstrated the effects of cell therapy with the improvement of several parameters, such as MNCV, SNCV, SNBF, and IENFD, using diabetic rats, we could not confirm the efficacy of cell therapy for peripheral nerve morphology because these rats with relatively short diabetic duration did not express morphological abnormalities except for axonal circularity [9]. The present study evaluated whether or not DPSC transplantation could ameliorate diabetic polyneuropathy in older animals with long-term diabetes accompanied by morphological deficits in the peripheral nerves.

We previously demonstrated that DPSCs highly expressed angiogenic and neurotrophic factors including NGF, NT-3, vascular endothelial growth factor (VEGF), and bFGF in vitro [14]. NGF expression levels were substantially lower in the long-term diabetic rats. Interestingly, DPSC transplantation significantly increased NGF expression only in the diabetic rats. Muscle biopsies contain intramuscular nerve endings, Schwann cells, vascular endothelial cells and connective tissue, in addition to muscle fibers. NGF is mainly synthesized in Schwann cells and is selectively trophic for sympathetic ganglia and small-fiber sensory neurons [18, 19]. We consider that Schwann cells would predominantly be affected by the long-term duration of diabetes, which contributed to the reduction in gene expressions of neurotrophic factors in skeletal muscles. Therefore, the physiological improvements made by DPSC transplantation might be mediated, at least in part, through the supply of various neurotrophic factors such as NGF and NT-3. It is possible that bFGF and VEGF secreted by the transplanted DPSCs also contribute to the improvement in nerve conduction velocities since neurosupportive ability has been reported in bFGF and VEGF [20].

Decreased IENFD in the footpad indicates a small-fiber abnormality in diabetic polyneuropathy [21]. DPSC transplantation significantly ameliorated the decrease in IENFD in the long-term diabetic rats, which is consistent with our previous observations in short-term diabetic rats [12]. The in vitro study showed that DPSC-CM promoted DRG neurite outgrowth, suggesting that secreted trophic factors from DPSCs contributed to the increase in IENFD in vivo. We further evaluated the sensory nerve function as CPT. The pulses at 2000, 250, and 5 Hz mainly stimulate large myelinated (Aβ), small myelinated (Aδ), and small unmyelinated (C) fibers, respectively, not only in humans but also in animals [22, 23]. Although hypoalgesia was observed in these nerve fibers in the long-term diabetes rats, all were improved by transplantation of DPSCs; this indicates that the efficacy of DPSC transplantation extended from small nerve fibers to large nerve fibers even in the long-term diabetic rats.

The morphometrical characteristics of diabetic polyneuropathy include axonal atrophy, nerve demyelination, and reduced regeneration of peripheral sensory nerve fibers [24]. The maturation of regenerated fibers is reported to be impaired after nerve injury in STZ-induced diabetic rats and BB/Wor rats [25, 26]. A previous study reported that STZ-induced mice exhibit thinning of the myelin sheaths, similar to human diabetic neuropathy, but these changes are not generally observed in STZ rats [27]. However, we found reductions in the fiber area, occupancy rate, myelin area, and myelin thickness, and an increase in the axonal-to-myelin area ratio of sural nerves in long-term diabetic nerves. Interestingly, DPSC transplantation significantly increased myelin thickness and myelin area. These results may indicate that DPSC transplantation predominantly leads to beneficial effects on Schwann cells. We previously demonstrated that a small number of the transplanted DPSCs differentiated into vascular endothelial cells, but not neuronal cells or Schwann cells in 12-week duration diabetic rats [12]. On the other hand, this study revealed that the secreted factors from DPSCs increased the Schwann cell viability and protein expression of MP0, which is crucial for Schwann cell development and peripheral myelin formation [16].

In 10–20 weeks duration diabetic animals, we and others demonstrated that the transplanted stem cells in skeletal muscles existed at least in part at the transplanted site for 4–8 weeks after transplantation [12, 28, 29]. Repeated transplantation was reported to improve the therapeutic efficacy [28], but it may inflict additional harm on patients. Since we have not examined how long the transplanted DPSCs exist in the long-term diabetic rats, further study will be required to address these issues.

Importantly, we and others found that the therapeutic efficacy was impaired when stem cells were prepared from diabetic or aged animals [30, 31]. Therefore, it is critical to secure stem cell sources from young individuals. For this purpose, DPSCs are an attractive candidate for cell therapy because they are easy to obtain from teeth extracted at an early age without further invasive procedures, unlike bone marrow or adipose tissue aspiration. Our previous study and this study demonstrated several mechanisms of the DPSC transplantation for diabetic polyneuropathy [14]. First, DPSCs express multiple secreted factors and DPSC-secreted factors improve neurons and Schwann cells. Furthermore, DPSC-secreted factors promote macrophage polarization towards anti-inflammatory M2 phenotypes, which result in the suppression of inflammation in the peripheral nerve of diabetic polyneuropathy. Second, some of the transplanted DPSCs still exist in the transplanted site and differentiate into vascular endothelial cells, suggesting that transplanted DPSCs cooperate with the resident cells and induce vasculogenesis. Understanding these therapeutic mechanisms of DPSC transplantation on diabetic polyneuropathy will provide insights into novel therapeutic strategies for elderly patients.

## Conclusions

We demonstrated that the transplantation of DPSCs induced neurophysiological and neuropathological recovery over a long duration of diabetic polyneuropathy. Secreted trophic factors from DPSCs, at least in part, contributed to the therapeutic effects on diabetic polyneuropathy. These findings indicate that the transplantation of DPSCs could be a promising therapeutic strategy for diabetic polyneuropathy.

## Additional file

Additional file 1: Table S1. Morphometric data of myelinated fibers in sural nerves. (DOC 44 kb)

## Abbreviations

bFGF: Basic fibroblast growth factor
BM-MNC: Bone marrow-derived mononuclear cell
CPT: Current perception threshold
DAPI: 4’,6-diamidino-2-phenylindole
DMEM: Dulbecco’s modified Eagle’s medium
DPSC: Dental pulp stem cell
DPSC-CM: Dental pulp stem cell-conditioned media
DRG: Dorsal root ganglion
EPC: Endothelial progenitor cell
FBS: Fetal bovine serum
FGF: Fibroblast growth factor
IENFD: Intra-epidermal nerve fiber density
LDPI: Laser Doppler perfusion image
MNCV: Motor nerve conduction velocity
MP0: Myelin Protein Zero
MSC: Mesenchymal stem cell
NGF: Nerve growth factor
NTF3/NT-3: Neurotrophin 3
SD: Sprague-Dawley
SNBF: Sciatic nerve blood flow
SNCV: Sensory nerve conduction velocity
STZ: Streptozotocin
VEGF: Vascular endothelial growth factor
