# Supplementary material for: Transplantation of dental pulp stem cells improves long-term diabetic polyneuropathy together with improvement of nerve morphometrical evaluation
Source: Stem Cell Res Ther. 2017 Dec 13;8:279. doi: 10.1186/s13287-017-0729-5 (PMC5729514; doi:10.1186/s13287-017-0729-5)
Supplement: Additional file 1: Table S1. — Morphometric data of myelinated fibers in sural nerves. (DOC 44 kb) [file 13287_2017_729_MOESM1_ESM.doc]

| **Additional file: Table 1.** Morphometric data of myelinated fibers in sural nerves | Axonal-to-myelin area ratio | 0.63 ± 0.06 | 0.61 ± 0.03 | 1.57 ± 0.39** | 0.58 ± 0.05^††^ | Data are mean ± standard error of the mean. ***P* < 0.01, **P* < 0.05 versus normal-vehicle rats; ^††^*P* < 0.01, ^†^*P* < 0.05 versus diabetic-vehicle rats. |
| --- | --- | --- | --- | --- | --- | --- |
|  | Axon Area (µm^2^) | 10.9 ± 0.7 | 10.5 ± 0.4 | 12.0 ± 0.9 | 9.3 ± 1.1 |  |
|  | Myelin Area (µm^2^) | 17.7 ± 1.1 | 17.3 ± 0.6 | 8.4 ± 1.7** | 15.9 ± 1.6^††^ |  |
|  | Occupancy rate (%) | 40.8 ± 1.2 | 41.3 ± 1.1 | 32.3 ± 2.1** | 42.5 ± 0.8^††^ |  |
|  | Density (fiber/mm^2^) | 14,380 ± 974 | 14,878 ± 550 | 15,269 ± 1,126 | 17,477 ± 1,936 |  |
|  | Fiber Area (µm^2^) | 28.6 ± 1.0 | 27.8 ± 0.7 | 21.4 ± 1.7* | 25.2 ± 2.6 |  |
|  | Limb | Normal-vehicle | Normal-DPSCs | Diabetic-vehicle | Diabetic-DPSCs |  |
